# End-to-End Teleoperated Driving Video Transmission Under 6G with AI and Blockchain

**DOI:** 10.3390/s26020571

**Published:** 2026-01-14

**Authors:** Ignacio Benito Frontelo, Pablo Pérez, Nuria Oyaga, Marta Orduna

**Affiliations:** Nokia XR Lab, 28050 Madrid, Spain; pablo.perez@nokia.com (P.P.); nuria.oyaga@nokia.com (N.O.); marta.orduna@nokia.com (M.O.)

**Keywords:** teleoperated driving, extended reality, 6G, blockchain, video quality, digital twins

## Abstract

Intelligent vehicle networks powered by machine learning, AI and blockchain are transforming various sectors beyond transportation. In this context, being able to remote drive a vehicle is key for enhancing autonomous driving systems. After deploying end-to-end teleoperated driving systems under 5G networks, the need to address complex challenges in other critical areas arises. These challenges belong to different technologies that need to be integrated in this particular system: video transmission and visualization technologies, artificial intelligence techniques, and network optimization features, incorporating haptic devices and critical data security. This article explores how these technologies can enhance the teleoperated driving activity experiences already executed in real-life environments by analyzing the quality of the video which is transmitted over the network, exploring its correlation with the current state-of-the-art AI object detection algorithms, analyzing the extended reality and digital twin paradigms, obtaining the maximum possible performance of forthcoming 6G networks and proposing decentralized security schema for ensuring the privacy and safety of the end-users of teleoperated driving infrastructures. An integrated set of conclusions and recommendations will be given to outline the future teleoperated driving systems design in the forthcoming years.

## 1. Introduction

Remote driving technology faces significant challenges in video transmission, network infrastructure, and hardware reliability. As scientists continue advancing our understanding of teleoperated systems, extended reality (XR) technologies are gaining traction in industrial applications, distinct from consumer metaverse developments.

Numerous previous studies have examined teleoperated driving (TOD) technology across various deployments and simulated scenarios [1,2,3,4,5,6,7,8]. Yu and Lee [1] demonstrate remote driving with real-time video over wireless networks and measure latency and control performance. Sato et al. [2] build a robust video transmission system over 5G/4G to reduce packet loss and jitter for remote driving. Nadir et al. [3] discuss immersive services over 5G and beyond, highlighting low latency, high bandwidth, and edge computing relevant to teleoperation. Taleb et al. [4] present a VR-based service management approach in B5G using a UAV control case, showing resource orchestration and QoS/QoE that can support remote driving. Hofbauer et al. [5] released Telecarla, an open-source CARLA extension for teleoperation research with off-the-shelf components. Den Ouden et al. [6] design and test a remote driving architecture on 4G/5G with on-road measurements of latency, reliability, and fail-over. Saez-Perez et al. [7] implement and validate a remote car driving framework on a commercial mobile network with KPIs for video quality and control responsiveness. Finally, Zhao et al. [8] survey remote driving, covering feedback, latency, support control, and real applications.

Despite this body of work, current studies often address video encoding, immersive interfaces, or network functions in isolation. However, safe and effective teleoperated driving requires integrating all three: high-fidelity video and rendering (for operator perception), immersive XR interfaces (to reduce distraction and present actionable cues), and resilient network functions (to meet strict latency, reliability, and security demands). Only by combining these technologies can a TOD system simultaneously achieve low end-to-end latency, and secure, prioritized data delivery under variable real-world networks.

This paper provides a comprehensive analysis of TOD deployment challenges through architectural measurements, quality of service (QoS) and quality of experience (QoE) studies, and novel deployment strategies for complete end-to-end solutions.

**Figure 1:**
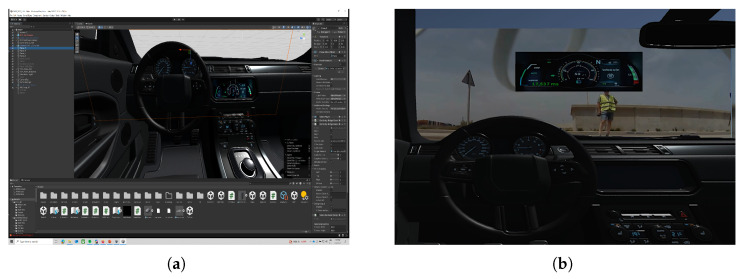
The XR application developed for TOD. (**a**) Unity 2022 Immersive XR Applications Running in Meta Quest 3 (**b**) Execution of the application combining telemetry and a high-quality inside view.

Our research utilizes an End-to-End XR Remote Driving solution developed across three real-world deployments: 5G-MOBIX [9], iNGENIOUS [10], and 5G+TACTILE [11]. This system presents a scalable architecture addressing critical remote driving challenges. To mitigate operator distraction [12], the system employs an immersive XR application that renders a photorealistic vehicle interior with real-time camera feeds. The implementation strategically uses planar video rendering to achieve realistic parallax effects, avoiding the inefficiencies of 360-degree stitched videos (Figure 1). We analyze multiple video encoding and presentation strategies, including 360-degree and 2D video formats, while considering real-world network constraints such as limited bandwidth, latency, and availability [13].

Building upon these deployments, this paper examines two critical areas: data (especially video) transmission optimization and network functions. We frame these areas as complementary: transmission and encoding strategies determine what visual information reaches the operator, while network functions ensure that this information arrives in a timely, secure, and prioritized way suitable for human-in-the-loop control.

Video quality represents a fundamental factor for teleoperated human drivers, making accurate quality quantification essential [14]. However, quality metrics should not be misinterpreted as operational feasibility thresholds. Beyond visual fidelity, TOD performance depends on the entire transmission pipeline: end-to-end latency, packet loss resilience, and encoder behavior under varying network conditions. Strict latency budgets (typically under 100 ms) and robust fail-over mechanisms are critical for maintaining safe operation when network quality degrades. While correlation between utility and quality may exist at higher bit rates, incorporating AI techniques from autonomous vehicle systems will provide more precise insights into remote driver safety when operating with re-encoded or degraded video signals. Assessment should therefore combine objective network and video metrics with task-level performance outcomes to determine practical operating envelopes for TOD.

Network functions encompass two fundamental challenges for teleoperated driving systems. Security and data authenticity require careful consideration for both stored and consumed information, where decentralization schemes and blockchain technologies offer powerful tools for integration into comprehensive solutions. Simultaneously, limited network resources necessitate strategic planning for 6G network deployment, incorporating concepts such as traffic prioritization, network slicing, and congestion management. These network function requirements directly impact the feasibility and scalability of TOD deployments, as they determine both the trustworthiness of transmitted data and the allocation of critical bandwidth and latency resources needed for safe remote operation.

Finally, a set of overall conclusions is established, with a special focus on the future research directions that should be taken to improve TOD systems.

## 2. 5G Deployments and System Architecture

Advanced networks (5G, 6G, WiFi) form the backbone of intelligent vehicle systems by enabling high-speed, low-latency data transfer needed for real-time tasks like traffic prediction, path planning, and cooperative control. 5G already supports URLLC and mMTC, connecting vehicles, infrastructure, and IoT devices. 6G is expected to offer higher speeds, greater bandwidth, tighter AI integration, and improved congestion management, enhancing adaptability for remote driving. The system architecture and real-world deployments are detailed in following sections.

### 2.1. Architecture

**Figure 2:**
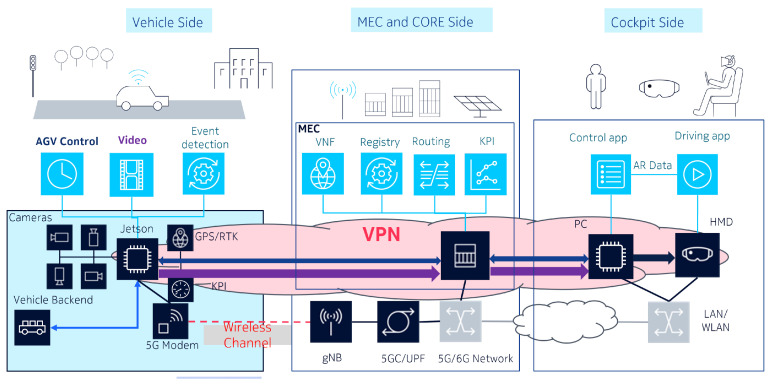
5G/6G Immersive Remote Driving Architecture. Purple arrows for video flow and blue arrows for control and telemetry flows.

The architecture of the entire system is divided into three main areas as depicted in Figure 2: vehicle side module, MEC/CORE and simulated cockpit application modules.

The main requirements for vehicle side modules in terms of hardware and software are low power consumption, light weight, and real-time capabilities. Another principal factor to take into consideration is using consumer products or at least inexpensive hardware. The NVIDIA Jetson Xavier NX low-power board is the main controller of the vehicle, making use of a video hardware encoder capable of absorbing and processing data from USB 1920 × 1080 30 FPS resolution cameras. Different bit rate encoding configurations are set depending on the camera orientation so that bandwidth usage is optimized and driving operation is carried out safely [15]. This setup connects to a 5G modem via a 1 Gbps Ethernet link.

With regard to software, it can be divided into control and video applications. The main duties of the control application on the vehicle side are connecting to the inner control layer of the vehicle which runs a Robotic Operating System (ROS) layer abstracting as well as enabling the inter-operation of all the involved modules.

Video acquisition and encoding are performed using customized video pipelines of four parallel video encoders available on the Jetson Xavier NX board with modified kernel capabilities. The streaming flows are sent in UDP with almost no buffers, so that video frames leave the vehicle as fast as possible. GPS/RTK and Depth Cameras were also present to provide precise positioning and simple obstacle detection for vehicle activities.

When designing the cockpit, it is observed that the typical setup for remote driving cockpit stands is composed of three screens and a lab network setup. The intention in this experimentation is to go one step beyond: prepare an immersive environment that includes real-time video working on a mobile network.

### 2.2. Deployments

Three real-life deployments of TOD are presented in the following section. Optimization of network transmission and encoding pipelines is present in all of them. Digital twin technology is also used to mitigate the case of low-bandwidth communications.

The solution for each deployment was validated, in terms of user experience and feasibility of the TOD activity, by pilot trials on each system’s regular operating conditions. Additionally, the main components of TOD QoE were considered, according to ITU-T GSTR-5GQoE [16]: end-to-end latency, quality of the video signals and quality of the sensor information. Those were combined to provide a synthetic QoE score using a parametric model [17], which operates in a similar way as ITU-T standard planning models [18].

#### 2.2.1. 5G-MOBIX Network Issues in the Context of Border and Roaming

This scenario is the most challenging. The purpose of 5G-MOBIX was to evaluate and analyze Cooperated, Connected, and Automated Mobility (CCAM) experiences with a 5G network country roaming process using immersive remote driving.

**Figure 3:**
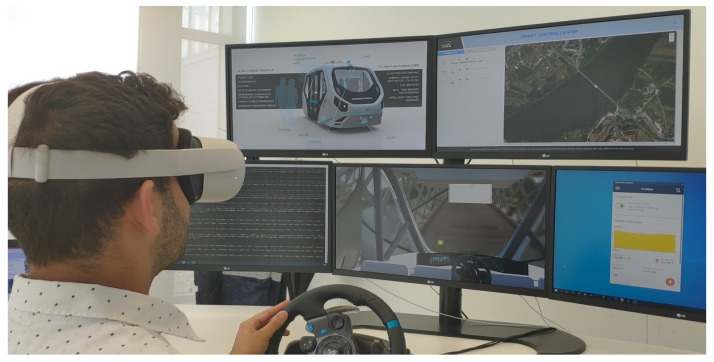
Remote Driving a Shuttle on the Spain–Portugal Border with real 5G Communications.

The Non-Standalone (5G) network was deployed in Spain and Portugal, sharing the commercial anchoring LTE on the Spanish side with a 5G operator. A 3.5 GHz band was used for the 5G part, while the 2600 MHz band was used for LTE anchoring. A first evolution of the immersive XR remote driving application was successfully evaluated on a closed track and on a real road with a shuttle. The remote driver was monitoring the autonomous driving process until a problem occurred. All this happens in the control center, as can be seen in Figure 3.

When an obstacle is in the road, a request for control appears in the mixed reality application through a blinking dialog in an overlay layer on top of the video. Once the vehicle takes over and grants control, the remote driver has full control of the shuttle to maneuver and avoid the obstacle. Finally, another return control request is sent back, and the vehicle resumes autonomous driving.

Round-trip time (RTT) is rendered every 100 ms in the augmented reality (AR) dashboard, indicating the time in milliseconds since the command was issued in the cockpit system to the moment at which it was received with the telemetry payload. The remote driver also has a map updated in the XR application showing the exact position of the vehicle while he/she is driving it. The haptic part of the setup is operated by a gaming steering wheel and pedals. The person feels like they are driving a normal physical car and acts with natural movements and reactions during the experience.

**Figure 4:**
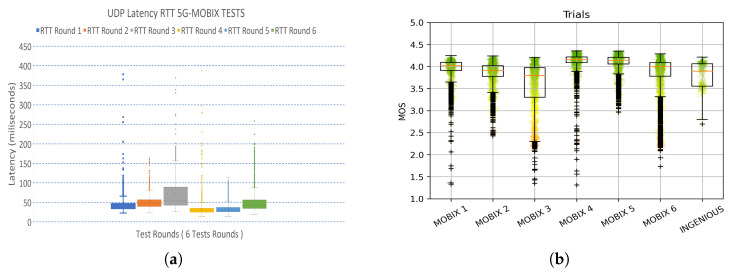
RTT latencies measured in 5G-MOBIX deployment and mean opinion score (MOS) subjective mapping. (**a**) RTT in milliseconds for control channel. (**b**) MOS according to the mapping from the model [17].

More than twenty driving sessions occurred, with durations ranging from a few seconds to 30 min using the immersive remote driving system without problems measuring RTT and related quality levels (Figure 4). The vehicle had an automatic protection system so that if it did not receive any control command for more than 300 ms, it would automatically stop.

#### 2.2.2. iNGENIOUS, the Internet of Things (IoT) Experience Different Haptic Devices

**Figure 5:**
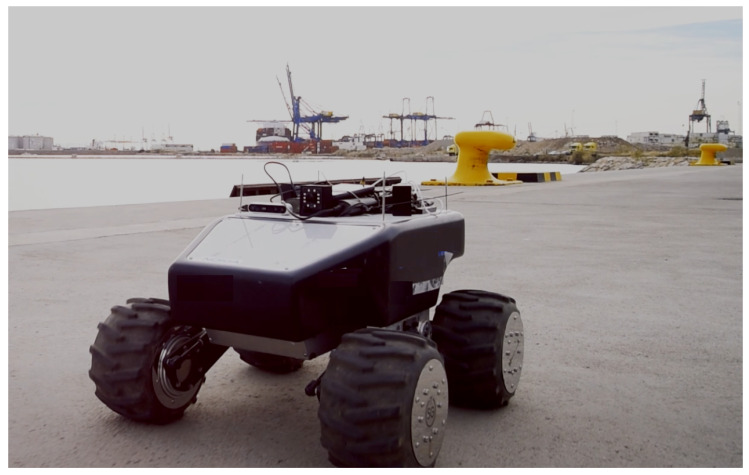
ROS AGV SummitXL handled in Ingenious project.

An evolved version of the XR application was evaluated in a 5G NSA n258 band deployment aimed at driving all types of Automated Guided Vehicles (AGVs) in an industrial environment. Two AGVs were driven: one ROS-based AGV (see Figure 5), with its cameras managed by the Jetson Xavier NX board directly connected by Ethernet to the AGV board, and another one with a proprietary board to control the movement.

**Figure 6:**
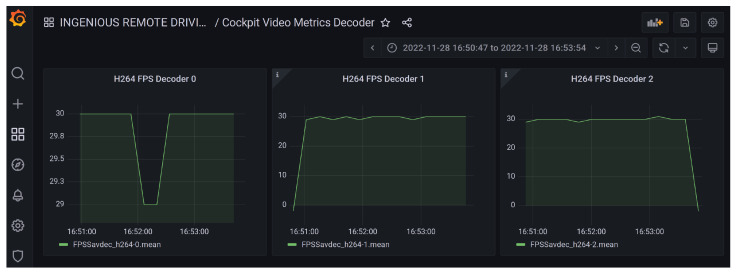
Decoded frames per second in immersive device as rendered by Grafana system.

Enhanced key performance indicator (KPI) collection capabilities (see Figure 6) and different hardware abstraction layers in the vehicle NodeJS application running on the Jetson board were created to be able to carefully monitor all possible network and application data on a graphical dashboard.

The variation in power received in the modem was more sensitive to slight changes in the orientation of the vehicle to the beam of the transmitting antenna. This led to stability issues. However, the available bandwidth was better than in the 3.5 GHz band, offering better maximum throughput under ideal conditions.

An improved version of the XR application was used. In this case, a photorealistic car interior and a new integrated dashboard were used. Having a more realistic and immersive model in the XR application resulted in a more comfortable driving experience, which could be executed during longer periods of time without the remote driver being tired. The ability to place the driver point of view in various positions in the virtual car added an additional level of ergonomics for the human driver. The AGV executed pre-programmed missions, moving to fixed points in the circuit. The remote driver started the XR session in supervised mode. When an unavoidable obstacle was in the middle, the AGV requested the remote driver’s control, and the XR session was established in driving mode, allowing the obstacle to be avoided. Then, a request was sent to resume the mission and the next point in the recorded path was reached while the remote driver continued the XR session in supervision mode. The round-trip time in the control plane is continuously monitored in both the KPI dashboard and the XR application, so that driving is absolutely safe.

#### 2.2.3. Haptic Responses—Working with Different Sensors and Actuators

**Figure 7:**
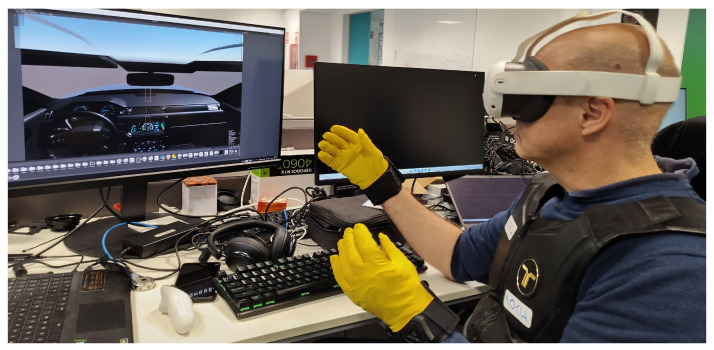
Using haptic gloves to control cockpit Unity application.

Haptic feedback provides tactile responses to user input, enhancing the interaction between drivers and AI systems. Vibrations and seat adjustments alert drivers to potential hazards or changes in system status, improving control accuracy. An example of this is the use of haptic gloves and chest as part of one of the deployments depicted in Figure 7.

#### 2.2.4. 5G-TACTILE—Digital Twins as Efficient TOD Representation

Bandwidth limitation or variability can be frequent issues when dealing with mobile networks. In this case, transmitting video over the network to the remote driver might not be feasible. Digital twins [19] are a solution for this kind of scenario, especially in the cases in which the driving environment is supposed to be immutable and there are no additional vehicles that are not under the control of the operator.

A realistic 3D model of the environment will be created in a VR application that renders it in real time.

As the position of the vehicle is known and can be transmitted in real time to the VR application running the digital twin, the vehicle can be perfectly driven by using a second-person perspective of it. The telemetry with Global Positioning System (GPS) position will then be mapped to the VR scene, providing a convenient way to drive the AGV (in this case) in a safe manner.

The GPS receiver we used is the EMLID REACH M2, which supports RTK, so we benefited from this feature in the project. This GPS creates a Wi-Fi hotspot that the AGV must connect to in order to receive the GPS signal, and the reading is performed through ROS. The positioning of the accuracy device is described in Table 1.

Regarding latency, we rely on the control channel of the AGV. In this case, we have the data for round-trip time which is an upper bound of the latency. This means that the latency in the update of the position will be this value in the worst case. The capture data for a typical driving session provides latency values under 50 ms (see Table 2).

**Figure 8:**
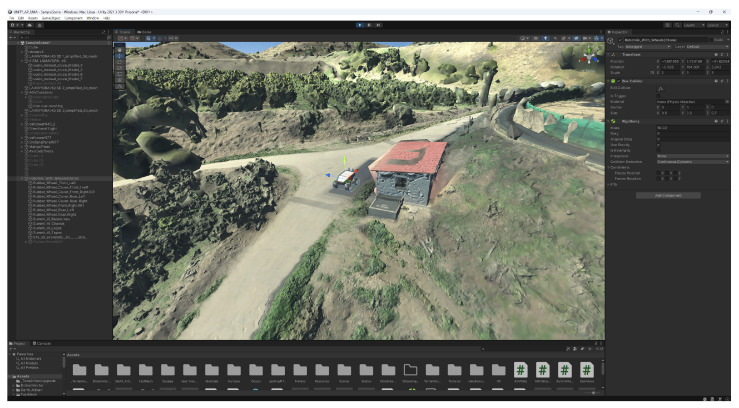
Digital twin of 5G-Tactile with mapped real-time robot position.

The digital twin model was created using two sources. The first source came from a photogrammetry flight in which several pictures were taken. These pictures were processed using Pix4Dmapper. Finally the model was exported in FBX format and then imported using Unity 2021, in which the business logic was implemented to be able to move the AGV in real time (Figure 8).

The second source comes from using Google Earth data combined with photogrammetry data. At this point, a reference point is established as the reference by selecting it from the Google Maps application and taking the exact coordinates’ location. The same point is located in the world being used in the Unity application and also selected as a reference for all local coordinates. After that, the scale is computed on both X and Y axes by selecting a second point and measuring the distance according to Google Maps coordinates and measuring the distance in terms of local units. With this scale we allow the movement of the AGV in the virtual world. Using gravity, rigid body and mesh collider features in the Unity model, we are able to allow the AGV to move using simulated physics in the mapped terrain.

## 3. Latency, Video Quality, and Control Systems in TOD

As outlined in the previous section, ITU-T identifies three main components of QoE for TOD: end-to-end latency, video signal quality, and sensor information quality. This section provides a comprehensive analysis of video processing and encoding challenges in remote driving systems, examining each of these critical components in detail. The investigation begins with latency considerations, including the measurement and characterization of end-to-end delays that directly impact driver reaction times and vehicle safety. Subsequently, advanced video processing techniques are explored, including predictive image transformation and 360-degree stitching methods that can enhance driver immersion while managing computational overhead. The section then examines the relationship between video quality metrics and AI-based object detection performance, revealing how traditional entertainment-focused quality assessments may inadequately predict safety-critical detection capabilities at low bit rates. Finally, customized control protocols and telemetry systems designed to minimize communication delays in safety-critical scenarios are presented. Together, these investigations provide essential insights for optimizing video transmission chains while maintaining the quality and responsiveness required for safe teleoperated driving operations.

### 3.1. Latency Considerations

Reaction time is crucial in both conventional and remote vehicle operation [20]. This section explores latency in transmission and encoding systems.

End-to-end (E2E) latency (L) is defined as the time elapsed from when a relevant visual event occurs in the real world until the remote driver’s reaction takes effect in the vehicle. Multiple factors contribute to this latency throughout the system, as detailed in Table 3.

The latency calculation follows this equation:

(1) $$\begin{array}{cc} L & =T_{c}+T_{e}+\left(\frac{B_{e}}{B_{t}}\right)\left(\frac{1}{\mathrm{FPS}}\right)\cdot 1000+T_{d}+T_{r}+T_{\mathrm{RTT}}+T_{h}+T_{x} \end{array}$$

The terms in Equation (1) can be grouped into three different factors: video latency ($T_{v}$), network round-trip time ($T_{\mathrm{RTT}}$), and reaction time of the human and vehicle ($T_{rx}$):

(2) $$\begin{array}{cc} L & =T_{v}+T_{\mathrm{RTT}}+T_{rx} \end{array}$$

This research focuses on measuring and characterizing ($T_{\mathrm{RTT}}$) and ($T_{v}$), while the reaction time factor, which examines differences between remote and local driving, falls outside the scope.

Since latency represents distance divided by speed, relationships between latency and speed for fixed distances can be established to determine distance uncertainty due to latency. This end-to-end latency defines distance uncertainty as the spatial distance the remote vehicle travels during the latency period (*L*) before the user’s reaction reaches the vehicle. This concept is crucial for assessing driving feasibility in next subsection.

#### 3.1.1. Video Latency Measurement

A direct gigabit Ethernet switch connection was used to measure latency from capture, encoding, decoding, and visualization factors. The experimental setup involved pointing a camera connected to a Jetson Orin NX at a computer displaying a millisecond chronometer. The camera connected to a 1 Gbps switch, with a PC on the same switch receiving, decoding, and rendering video at 60 Hz refresh rate.

Over 100 samples per experiment were collected using two approaches:

1. Direct MJPEG transmission via RTP/UDP.
2. MJPEG flow re-encoded to H.264 using NVIDIA hardware encoder on Jetson Orin NX, configured at a 4 Mbps bit rate, and transmitted via RTP/UDP.

The stream was received, decoded and rendered in a PC. Latency measurement involved capturing UDP packets using tcpdump and averaging received bits over 10 s periods. The encoder design using Constant Bit Rate (CBR) ensures minimal bit rate variations, avoiding network peaks. This allows us to work with a small number of samples.

The following gstreamer pipelines were used for transcoding, MJPEG decoding, and H.264 decoding, respectively:

*gst-launch-1.0 v4l2src device=/dev/video0 io-mode=4 do-timestamp=true ! "image/jpeg, width=(int)1920, height=(int)1080, pixel-aspect-ratio=(fraction)1/1, framerate=(fraction)30/1" ! jpegdec ! nvvidconv ! nvv4l2h264enc iframeinterval=30 bitrate=4000000 insert-sps-pps=1 ! ’video/x-h264, stream-format=(string)byte-stream’ ! rtph264pay mtu=1400 ! udpsink host=192.168. 1.65 auto-multicast=true port=5002*

*gst-launch-1.0 -v udpsrc multicast-group=1port=2 caps="application/x-rtp, media=(string) video, encoding-name=(string)JPEG" ! rtpjpegdepay ! jpegdec ! autovideosink*

*gst-launch-1.0 -vvvvv udpsrc multicast-group=1port=2 auto-multicast=true ! application/x-rtp ! rtpjitterbuffer latency=10 ! rtph264depay ! h264parse ! nvh264dec ! videoconvert ! autovideosink*

**Figure 9:**
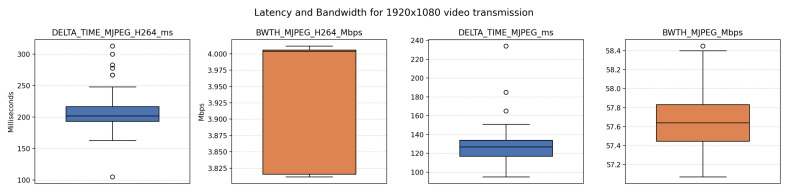
Latency and bandwidth consumption for 1920 × 1080 camera.

The experiment revealed that while MPEG provided approximately 100 ms lower latency, it required nearly 14 times more bandwidth (57 Mbps for MJPEG vs. < 4 Mbps for H.264/Advanced Video Coding (AVC)). This results in 171 Mbps uplink requirements for the MJPEG scenario (Figure 9). Statistical analysis of the collected data is presented in Table 4.

**Figure 10:**
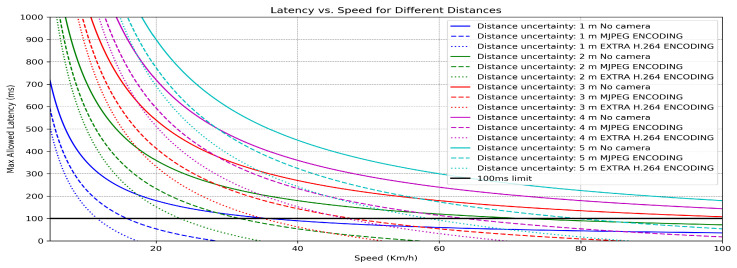
Allowed latencies for different speeds and encoding schema.

Finally, the influence of latency on distance uncertainty, or on the possible maximum error, can be analyzed by considering the presentation of past images. In the graph below, the solid-colored curves indicate the latency associated with a particular combination of distance uncertainty and speed, assuming pure visual perception without any camera involvement. These baseline curves are then modified by subtracting the mean latency recorded in the experiment summarized in Table 4. This operation yields two additional curves located beneath the original solid curves, representing the remaining latency budget that can be attributed solely to network-induced delay. Furthermore, a horizontal reference line at 100 ms is included to evaluate whether any given curve surpasses this limit. If a curve remains above the reference line, it implies that driving with the specified uncertainty at the corresponding speed is still achievable with network-induced latency (Figure 10).

#### 3.1.2. RTT Measurement

The second factor in the latency equation is the round-trip time $T_{RTT}$. RTT represents the time for data to travel from cockpit to vehicle plus return time. The implemented control protocol sends JSON payloads with unique command identifiers via UDP from cockpit to vehicle, receiving acknowledgment responses with the same identifier plus telemetry data. Millisecond-precision timestamps for both events calculate the difference. No NTP synchronization is required since packet departure and arrival times are measured using the local clock, providing an upper bound for uplink network latency.

### 3.2. Predictive Image Transformation and Stitching

**Figure 11:**
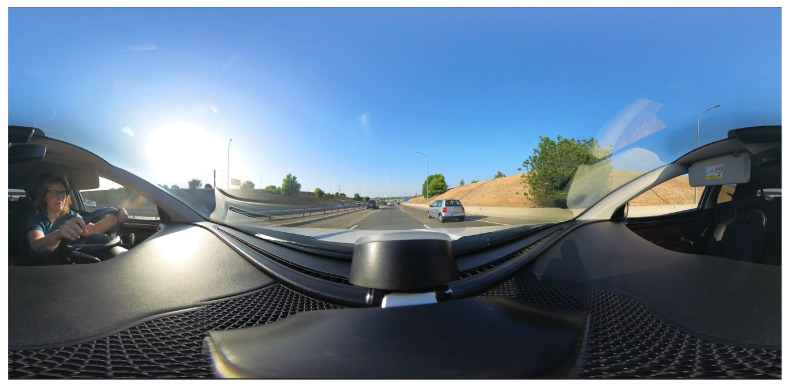
Post-processed 360-degree video frame captured with a camera placed in car driving on a motorway.

Processing multiple video sources into 360-degree format [21,22,23] adds latency to the transmission chain for high-quality stitching (Figure 11). Specialized hardware and AI can improve processing times.

**Figure 12:**
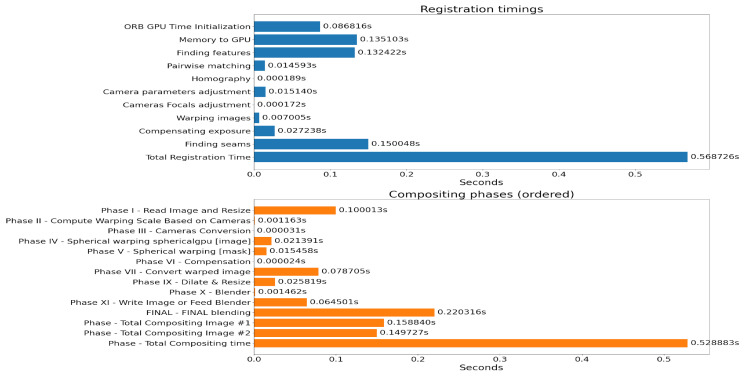
Stitching phase duration using high-quality stitching of two 4K-resolution images running in RTX 3070 GPU.

Analysis reveals several phases of preparing compound 360-degree images with quality stitching. High-quality stitching of two 4K images using Graphics Processing Unit (GPU) processing requires approximately one second (Figure 12).

**Figure 13:**
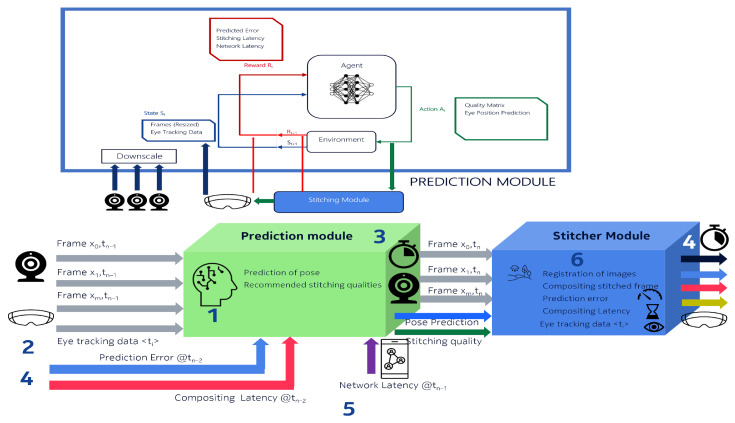
Proposal for smart stitching aided by AI using reinforcement learning and HMD movement prediction.

Advanced techniques leveraging edge computing and AI, particularly reinforcement learning, enable parallel and predictive stitching based on remote driver gaze direction. The intelligent stitching architecture (Figure 13) executes all phases within a dedicated module supported by reinforcement learning-based convolutional networks that predict gaze movements and dynamically prioritize image processing tasks.

### 3.3. Video Quality and AI Object Detection, Degradation with Bit Rate, Relationship with Autonomous Driving

Bandwidth constraints are a significant challenge in video transmission. To optimize data usage, re-encoding techniques reduce bit rate without sacrificing quality. For example, dynamic bit rate adjustment ensures that videos adapt to network conditions, maintaining clarity while minimizing bandwidth consumption.

The quality of the video directly impacts the utility of intelligent vehicle systems. High-quality video ensures accurate object detection, real-time monitoring, and effective communication between devices. However, balancing resolution and bit rate is essential to maximize system efficiency under varying network conditions. The complexity metrics of spatial/temporal information (SI/TI), VCA [24], and EVCA [25] can be assessed based on the percentage of degradation, while traditional video quality assessments (VQAs), such as peak signal-to-noise ratio (PSNR), video multimethod assessment fusion (VMAF) [26] (using the same model across all resolutions), Rec. ITU-T P.1204.3 [27], and COVER [28], can be measured using their respective raw scores. For YOLOv7 [29] object detection, degradation can also be quantified as the percentage of detection capability at a specific bit rate relative to the original master quality (e.g., CARS_PCT for cars). Similarly, lane detection degradation (LD_PCT) is calculated as the mean intersection over union (MIoU) between the detected lane pixels in the degraded image and those in the master quality using YOLOPV2 [30]. Mathematically, this is expressed as follows:

(3) $$\mathrm{LD\_PCT}=\frac{\mathrm{Intersection}\;\mathrm{of}\;\mathrm{Masks}\;\mathrm{for}\;Q1\;\mathrm{and}\;Q2}{\mathrm{Union}\;\mathrm{of}\;\mathrm{Masks}\;\mathrm{for}\;Q1\;\mathrm{and}\;Q2}$$

where Q1 is the binary mask corresponding to the first quality level (bit rate) and Q2 is the mask for the second quality level.

The correlation between state-of-the-art quality metrics and AI metrics has been computed for six different autonomous driving datasets [31,32,33,34,35,36] to check if there is some correlation between standard video QoE metrics used for entertainment and the degradation of detection as executed by an AI process. Ten-second sequences were used as input from the source datasets. The short-duration clips were encoded with ITU-T H.264/AVC: libx264 (x264 core 163 r3060 5db6aa6) using FFmpeg version 4.4.2-0ubuntu0.22.04.1.

**Figure 14:**
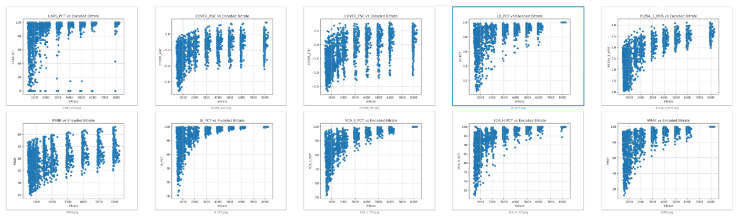
Degradation of video quality metrics and object detection (blue box) with video encoding.

Eleven different bit rates were selected to create different quality levels ranging from 8 Mbps to 0.5 Mbps. This set of 216 sequences was the input to the objective video quality metrics and AI algorithms calculation, leading to 2376 encoded video results. Then, the video quality metrics are calculated in total with the detection metrics of different objects significant for the driving operations and graphed for trend indications as seen in Figure 14. All the data is publicly accessible at the github repository mentioned in Data Availability Statement.

**Figure 15:**
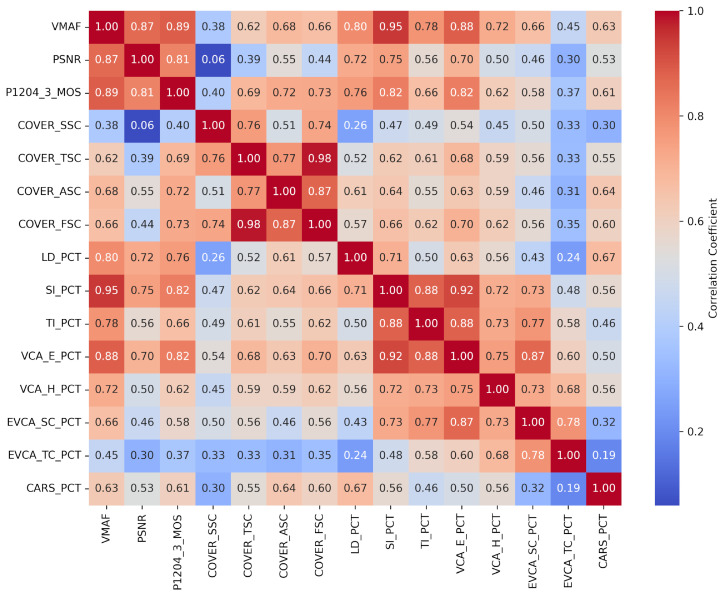
Correlation matrix of video quality metrics and object detection for all datasets.

It is interesting to check whether there is some correlation between the object detection metrics (LD_PCT: lane detection; CAR_PCT: car detection) and the video quality metrics. To do this, Pearson correlation is calculated by comparing the different data variations across bit rates. A moderate-to-high correlation was found for some specific metrics as seen in Table 5 and the correlation matrices depicted in Figure 15.

The statistical *p*-values for the data presented in Table 6 are very low, indicating that the observed results are highly unlikely to have occurred by random chance. Furthermore, the 95% confidence intervals (CI95) for the selected data are reported in Table 7.

**Figure 16:**
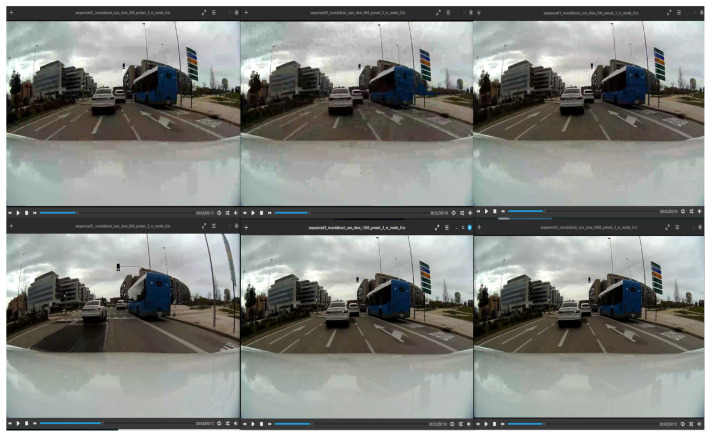
Driving video re-encoded at different bit rates. From left to right and top to bottom: 300 Kbps, 400 Kbps, 500 Kbps, 800 Kbps, 1 Mbps, 2 Mbps. Limit of 500 Kbps could be considered safe in this particular example.

In some occasions, bandwidth offered to the vehicle in the uplink connection will be limited to bit rates as low as 1 Mbps. It is important to question whether this quality is enough for safe remote driving of a vehicle, as shown in Figure 16. Standard video quality metrics will not provide the researcher with an answer to this question, as such metrics were designed to predict the opinion of an average user viewing entertainment content.

**Figure 17:**
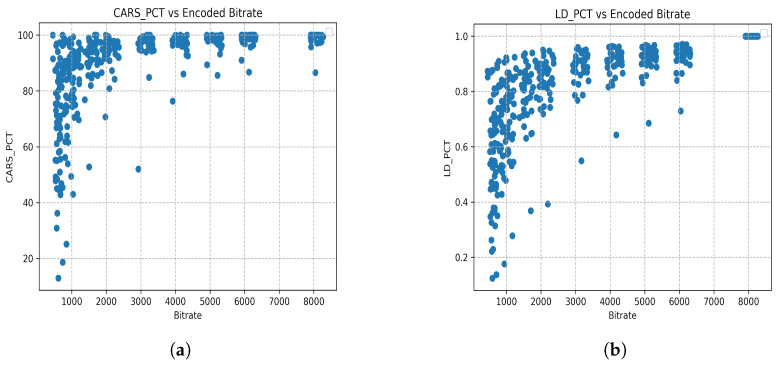
Degradation of level of car and lane detection when re-encoding at different bit rates. (**a**) Degradation of car detection using YOLOV7 when re-encoding at different bit rates from 300 Kbps to 8 Mbps. (**b**) Degradation of lane detection using YOLOPV2 when re-encoding at different bit rates from 300 Kbps to 8 Mbps.

It is more important to pay attention to the degradation of the detection of objects (see Figure 17) when video is being re-encoded at these really poor bit rates. Higher PSNR values (or bit rate values) do not necessarily mean that segmentation or object detection works proportionally better [37,38,39].

### 3.4. Control Protocols and Telemetry

**Figure 18:**
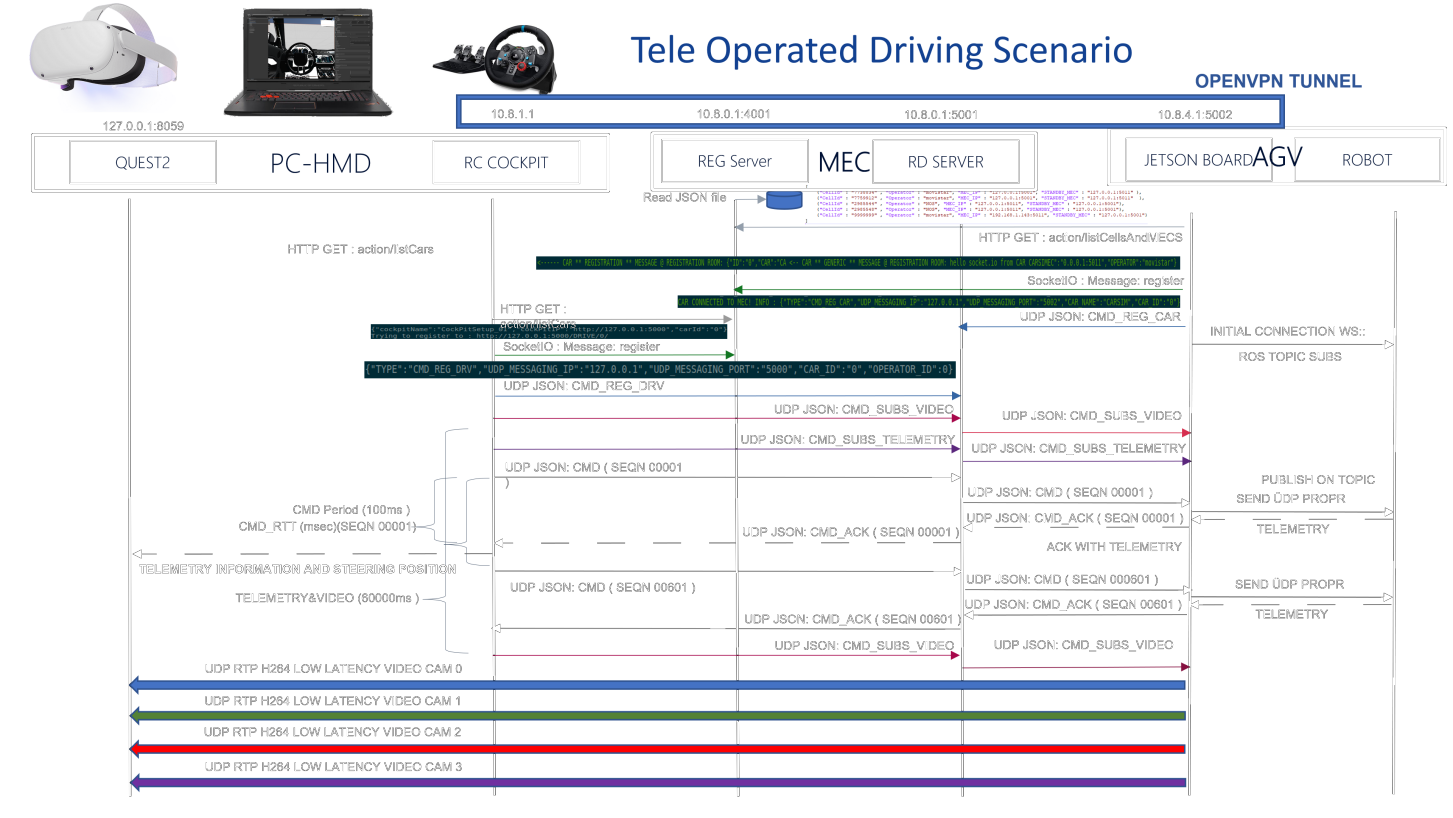
Proprietary control to send and receive actuation and telemetry for TOD.

A customized protocol has been developed to optimize control data transmission latency for teleoperated driving applications. The system uses UDP numbered messages with corresponding ACK responses, measuring RTT for every command without retransmission. Commands are sent every 100 ms, with emergency protocols triggered when ACK delays exceed 300 ms or messages are lost, initiating emergency stops or autonomous mode activation (Figure 18).

## 4. Network Functions for TOD

The preceding sections have primarily focused on the data itself and its acquisition, processing, quality, and the application of artificial intelligence to optimize its utility. In the context of real-world deployments, however, network functions play a critical role in bridging the remote control system and the vehicle. These functions are tasked with ensuring data integrity while providing a communication channel characterized by high capacity, low latency, and robust security, all of which are essential for the demanding requirements of remote driving.

This discussion will explore how key performance indicators (KPIs) and telemetry data can be safeguarded against malicious attacks through blockchain-based strategies. Additionally, we will examine how advanced 5G capabilities, such as the Low-Latency Low-Loss Scalable Throughput (L4S) feature, can enhance network efficiency while minimizing packet loss. To validate these approaches, prototypes and laboratory deployments will be utilized to demonstrate the feasibility of integrating blockchain and L4S technologies into potential real-world implementations.

### 4.1. Key Performance Indicators and Sensitive Data

Telemetry and control data are critical to ensuring authenticity and reliability in intelligent systems. Compromised telemetry, whether through theft or replacement, can lead to significant privacy and service issues, as unauthorized entities may store or distribute these data in violation of privacy regulations, which vary depending on local legislation. Similarly, the replacement of control data with falsified information poses serious risks to safety and service quality. To mitigate these risks, robust mechanisms must be implemented to prevent such scenarios.

Decentralized storage offers a promising solution to maintain data integrity, accessibility, and security in intelligent vehicle networks. This section examines the application of blockchain technology for decentralized storage, focusing on two approaches: Ethereum-based solutions and ad hoc blockchain deployments. Blockchain, as a distributed ledger technology, securely records transactions across multiple nodes, offering key features such as immutability, transparency, and resistance to manipulation. Ethereum, a leading platform for Decentralized APPlications (dApps) and smart contracts, provides a robust ecosystem for developing blockchain-based solutions, including secure storage systems.

Two potential applications of blockchain technology are proposed. The first addresses the prevention of control data tampering or theft. Laboratory experiments have demonstrated the feasibility of this approach without negatively impacting the performance of remote driving operations. This method ensures the integrity of control data in real-time environments. The second proposal is theoretical and involves monitoring remote drivers’ KPIs and linking them to monetary compensation via smart contracts. This solution aims to address the challenges associated with telemetry data theft or replacement, offering a novel approach to incentivize accountability and reliability in remote driving operations.

#### 4.1.1. Using Ad Hoc Blockchain Linked to TOD Control Protocol

In the context of TOD, the adoption of ad hoc blockchain deployments presents a promising solution for addressing the unique requirements of this ecosystem. Unlike established platforms such as Ethereum, ad hoc blockchains are custom-designed to meet specific operational needs, offering greater flexibility in terms of functionality and integration. However, this approach also introduces challenges, particularly in ensuring robust security, achieving scalability to handle increasing data loads, and maintaining the system effectively over time. Careful consideration and ongoing research are essential to address these challenges and unlock the full potential of tailored blockchain solutions for TOD applications.

**Figure 19:**
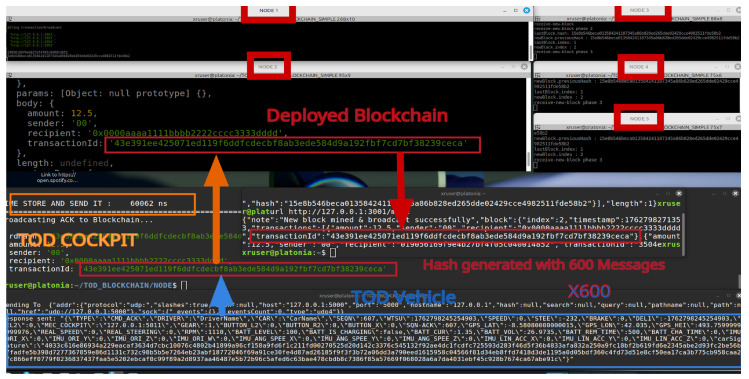
TOD system prototype communicating and storing data in a local blockchain implementation.

A prototype blockchain system for storing telemetry data in TOD applications has been successfully implemented. The system operates by collecting a set of telemetry data instances and generating a hash based on this dataset. The hash is then transmitted to a blockchain system with (n) nodes using the transaction/broadcast API. Upon receipt by one node, the hash is broadcast to the remaining nodes and queued as a pending transaction. Once the mining process is completed, the transaction is permanently recorded in the blockchain (Figure 19). Due to the immutable nature of blockchain technology, the hashed information remains secure and tamper-proof.

If a malicious actor attempts to falsify telemetry data, the resulting hash will differ from the authentic hash already stored on the blockchain. Tampering with the blockchain itself to insert fraudulent transaction ID hashes is also infeasible due to its inherent security features.

To verify the authenticity of data, the following steps are performed:

1. Generate a hash for the data in question.
2. Search for the hash within the blockchain.
3. If the hash exists, the data are verified as authentic; otherwise, the data are deemed fraudulent.

One challenge in this system is the latency associated with inserting data into the blockchain. To address performance concerns, telemetry data are packed into batches over a configurable time period. For instance, if telemetry data are collected every 100 ms and grouped into batches of 600 samples, a blockchain transaction can be issued approximately every 10 min. Experimental results indicate that generating hashes for 600 telemetry acknowledgment messages, creating the hash, and transmitting it to the blockchain consistently takes less than 100 ms. The system can be further optimized by adjusting the number of nodes and the frequency of data batching.

The timestamp associated with each transaction corresponds to the local time at which the telemetry and control data are transmitted to the blockchain. To ensure system reliability, the prototype was tested with a five-node blockchain, demonstrating its ability to prevent data tampering. Additionally, each vehicle can generate a digital signature for its data before storing it on the blockchain or another decentralized system. The hash function ensures that any alteration of stored data can be detected by comparing hashes, while encryption safeguards sensitive information during transmission and storage.

Several cryptographic methods and strategies are particularly suitable for TOD applications, including zero-knowledge proofs [40], threshold signatures [41], lightweight cryptography [42], post-quantum cryptography [43], and secure multi-party computation (MPC) [44]. These approaches involve a tradeoff between performance and security. Lightweight cryptography is ideal for low-power devices within vehicles but may be vulnerable to certain cryptographic attacks. Conversely, post-quantum cryptography methods, while requiring greater computational resources, can be implemented on edge devices with higher processing capabilities.

#### 4.1.2. Using Ethereum and Smart Contracts for Monetization of a Remote Driver Platform

**Figure 20:**
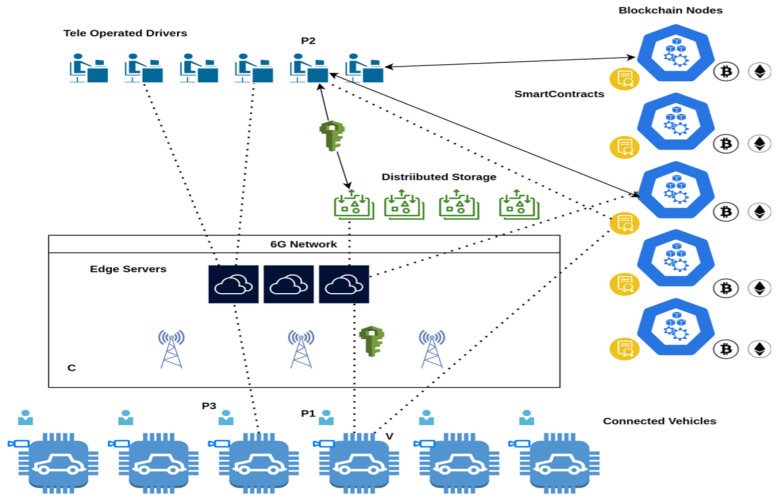
Distributed architecture for monetization of teleoperated driving.

Ethereum and smart contracts provide a foundation for a distributed architecture aimed at monetizing remote driving services, as illustrated in Figure 20.

In this framework, multiple participants (denoted as $P_{i}$) must establish mutual trust to ensure the system’s functionality. Specifically, $P_{1}$ must trust $P_{2}$ to deliver the service to the customer and facilitate payment, while $P_{2}$ must trust $P_{1}$ to ensure proper compensation for the service. Similarly, $P_{3}$ must trust both $P_{1}$ and $P_{2}$ to receive their share of the service-related compensation. All participants must also trust *C*, the communications infrastructure, to ensure reliable operation, which requires monetary compensation. Furthermore, all $P_{i}$ must trust the capabilities and operational state of the vehicle (*V*).

The core concept of this architecture is the use of immutable smart contracts to automate payments based on key performance indicators (KPIs) exchanged between the driver and the vehicle. These contracts ensure that payments are made according to the quality of service provided to the end-user. Failure to meet minimum requirements for security, timeliness, or comfort may result in penalties, such as reduced payments for the driver or the service provider.

The system is designed to handle KPIs asynchronously, with trusted data collected periodically (e.g., every five minutes), hashed, and stored on the blockchain for security. Retrieving this data within the smart contract does not interfere with the control mechanisms of TOD, as the data is processed in a non-blocking manner. However, executing transactions and achieving consensus within the blockchain requires computational and energy resources, which must be financed by the involved participants and potentially external entities, such as advertisers or public funding.

Algorithm 1 presents the pseudo-code for a simplified Solidity smart contract. In this example, a single oracleRelayer is used, which should be replaced with a more robust oracle solution, such as ChainLink, Band Protocol, Relic Protocol, or Redstone, to enhance reliability and security.
**Algorithm 1:** Vehicle KPI Payment & Penalty Contract**Data**: Deposit amounts, KPI reports, and user/driver info **Result**: Settlement of payments and penalties
initialize owner, oracleRelayer, platformFeeBps, trips, reports, withdrawable;
   Function createTrip(tripId, user, driver) external onlyOwner:
       requires trip with tripId does not exist;
       creates trip with user, driver, deposits = 0, settled = false;
       emits TripCreated;
   Function depositForTrip(tripId) external payable:
       requires trip exists and not settled;
       if sender == user: depositUser += msg.value;
       if sender == owner: depositCompany += msg.value;
       emits TripDeposited;
   Function subMitKpiReport(report) external onlyRelayer:
       requires trip exists and not settled;
       stores report and emit KpiReported;
       compute fees and penalties:
       calculate platformFee = userDeposit * platformFeeBps/10000;
       assign remaining user deposit to company;
       assign company funds proportionally to combinedScore;
       if combinedScore < lowThreshold:
           compute penalty to driver; adjust driver payment and penalty to user;
       if serviceFail:
           refund 80% of user deposit, reduce company payout, add penalty to user;
       update withdrawable balances for owner, driver, user, and platform;
       set trip as settled and emit Settled;
   Function withdraw() external:
       requires withdrawable balance > 0;
       transfer balance to caller;
       set withdrawable balance to zero;
       emit Withdrawn;
   Function receive() external payable:
       add msg.value to owner’s withdrawable balance;


### 4.2. Transport Optimization with L4S

This section analyzes how congestion control features can help in optimizing the video uplink traffic bit rate by providing control data to the end-user applications, specifically the video encoder ones. Detailed studies can be found correlating the bit rate frequency of L4S signaling and its use in XR applications, such as [45,46]. The L4S implementation in our lab will be described as well as real uplink video load testing of the future use and actual camera that will transmit video using UDP.

#### 4.2.1. L4S Feature Explanation and Implementation

Low latency is not provided by the network. Instead, low latency results from the careful behavior of the scalable congestion controllers used by L4S senders. The network does have a role, primarily to isolate the low latency of the carefully behaving L4S traffic from the higher queuing delay needed by traffic with preexisting classic behavior. The network also alters the way it signals queue growth to the transport. It uses the Explicit Congestion Notification (ECN) protocol, but it signals the very start of queue growth immediately, without the smoothing delay typical of classic AQMs. Because ECN support is essential for L4S, senders use the ECN field as the protocol that allows the network to identify which packets are L4S and which are classic. There are three main components to the L4S architecture: the scalable congestion control on the sending host, the AQM at the network bottleneck and the protocol between them.

**Figure 21:**
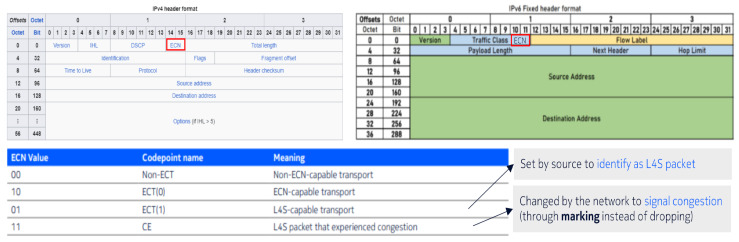
Congestion bytes in IPv4 and IPv6 headers.

First of all, it is important to understand that ECN and its marking are extensions of IP protocol as seen in the IP protocol headers (Figure 21).

ECN is an extension of the IP protocol. It allows end-to-end notification of network congestion without dropping packets. The ECN two-bit field is included in the IP header of the IP packet. The ECN field can have the following values:

- Value 01 ECT(1): The included packet is of type L4S and requires special attention.
- Value 11 CE:
  – When a node becomes congested, it starts marking incoming L4S packets (identified by ECN = 01) with a new value of 11 CE, indicating congestion. This process is called ECN marking.
  – The L4S packets with ECN = 11 are forwarded to the next nodes until they reach the receiver. The receiver, upon receiving an IP header with the ECN field set to 11, knows that congestion occurred in the network for that data stream.

One or more QoS flows may contain a mix of L4S and non-L4S packets. The gNB analyzes the IP headers to divide the data into an L4S stream and a non-L4S stream based on the ECN value. The Queue Classifier, which relies on the ECN value set in the IP headers, splits the packets into two streams:

- L4S packets (ECN = 01 ECT(1) or ECN = 11 CE) are directed to the L-Queue.
- Non-L4S packets (ECN = 10 ECT(0) or ECN = 00 Not-ECT) are sent to the C-Queue.

**Figure 22:**
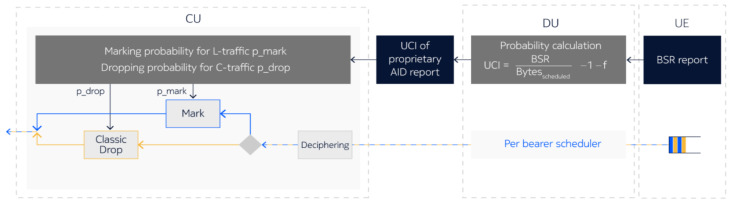
Smooth Marking Probability block used to adjust the marking probability to Prague Congestion.

L4S data handling relies on Buffer State Reports (BSRs) received from the UE, which inform the gNB about the uplink data volume waiting to be scheduled. The scheduling efficiency is based on the BSR and the amount of data scheduled, measured in Bytes_scheduled. The Smooth Marking Probability block is used to adjust the ECN marking probability in accordance with Prague Congestion Control (Figure 22). For every L4S packet, a uniform random number u is generated and the packet is ECN marked with ECN = 11 CE if u is less than p_mark. Similarly, the Smooth Dropping Probability block evaluates non-L4S packets; for each one, a uniform random number u is generated, and the packet is dropped if u is less than p_drop. Dropping of non-L4S packets is supported when the parameter l4sUlLatencyShield is set to true. This approach helps to manage congestion, as excessive C-traffic can increase latency not only for C-traffic itself but also for L-traffic.

When all the data reported by the BSR has been scheduled, the ratio BSR/Bytes_ scheduled equals 1. If only part of the BSR-reported data is scheduled, this ratio increases. The higher the BSR/Bytes_scheduled ratio, the more the congestion level for uplink transmission is considered to be determined by the gNB DU. The congestion level is communicated from the DU to the CU via a Congestion Information (UCI) element within the F1U: Assistance Information Data (AID) message. Based on the received UCI, the gNB-CU evaluates the marking probability for L4S packets and the dropping probability for non-L4S packets.

**Figure 23:**
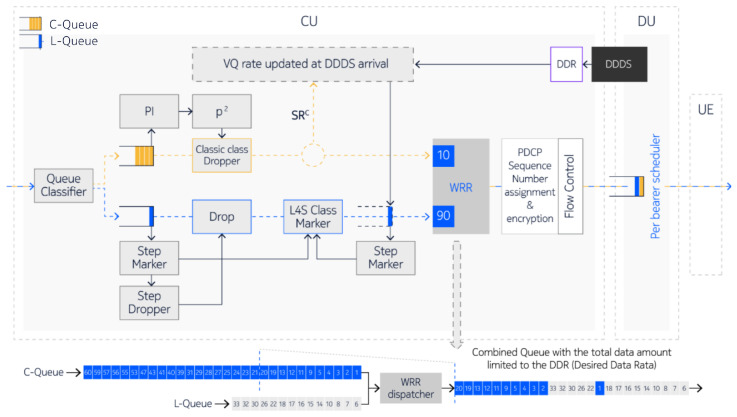
gNB architecture using separate queues for L4S and legacy packet control.

Incoming packets are split into either the C-Queue or the L-Queue. Packets in the C-Queue are dropped using a PI2 algorithm. The Step Marker and Step Dropper within the L-Queue monitor the delay of the queue. If the L-Queue delay exceeds the threshold set by a variable called 4sStepLatencyThreshold, the Step Marker performs ECN marking by setting the ECN field to 11 CE for incoming IP packets. When the delay surpasses the higher threshold specified by l4sDropLatencyThreshold, the Step Dropper drops the incoming IP packets. The Weighted Round-Robin (WRR) dispatcher merges both queues, prioritizing the L-traffic with a 90% priority to ensure L4S packets are moved to the front of the combined queue. The total data volume in this combined queue is limited to the reported DDR multiplied by the DDDS message periodicity (see Figure 23).

#### 4.2.2. L4S Tests Evaluating Congestion Counters in UDP Traffic Saturation

Tests in a 5G-Advanced lab network have been performed using L4S signaling bits. The idea of the test is related to TOD in the sense that an adaptive rate video encoder wrapper has been implemented to allow awareness of the congestion level in the network and re-provision the desired output bit rate (see sample source code in Listing 1.

**Listing 1.** Code showing how to change bitrate in real-time in gstreamer managed video
encoder.

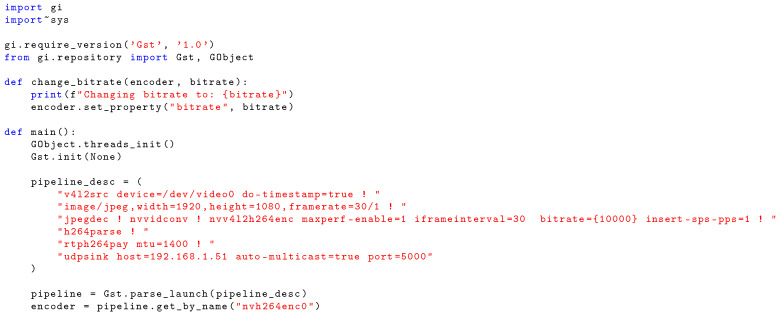



To perform the experiment, we designed an artificial step excitation into the network produced by one UE mobile network user. The smart video encoder receives the saturation in the network thanks to the L4S feature sending feedback about the congestion level. Then, the encoder reduces its target bit rate to use less bandwidth.

We focused on the uplink testing strategy due to the fact that TOD scenarios imply UDP uplink traffic from cameras connected to mobile networks. The process involved an L4S stream source operating over UDP with a variable bit rate ranging from 10 to 20 Mbps, initially starting at 20 Mbps. During testing, if congestion was detected at the receiving end, the source would decrease the bit rate; conversely, if no congestion was observed, the bit rate would increase. The L4Stream sink in UDP monitors congestion levels, reporting any detected congestion back to the source every second.

**Figure 24:**
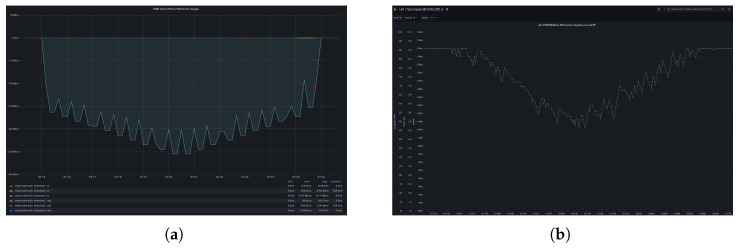
Network traffic injection. (**a**) Artificial traffic step function. (**b**) Adaptive encoder traffic.

Additionally, a UDP traffic generator produced traffic between 15 and 25 Mbps, following a Ramp-Up and Ramp-Down pattern—starting at 15 Mbps for 30 or 60 s, then increasing by 1 Mbps each period until reaching 25 Mbps, before decreasing by 1 Mbps per period back down to 15 Mbps. We can see in the left side of Figure 24 the artificial traffic excitation injected into the network. In the right side of Figure 24 we observe how the encoder progressively reduces the bandwidth being used adapting to the traffic shape of the excitation.

**Figure 25:**
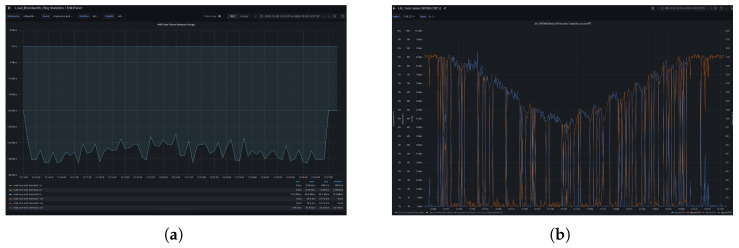
L4Scontrol monitoring. (**a**) Aggregated traffic in the radio uplink for the slice allocate for TOD video traffic. (**b**) Congestion level and ECN(01 and 11) counters.

We need to monitor the traffic with ECN bits, tracking the total bandwidth utilized in the UPF and assessing traffic loss as well as congestion reports during the testing process. The resulted overall traffic now is limited to the bounds in which there is no congestion and no traffic loss. In parallel, we are able to take note of the ECN counters and the congestion levels in Figure 25.

## 5. Conclusions

The integration of advanced technologies such as AI, blockchain, 6G communication, and digital signatures has transformed the development and application of modern systems. This paper examines various aspects of technological advancements, including data integrity, teleoperation, and secure communication, emphasizing the pivotal role these innovations play in addressing contemporary challenges.

### 5.1. Video Latency, Quality of Experience and Artificial Intelligence

A detailed analysis of the factors that are involved in video transmission for teleoperated driving makes us understand that latency is critical for remote operating a vehicle while the video quality is not as relevant as for the case of entertainment use case. Using state-of-the-art AI object detection techniques will help us correlate and infer which is the appropriate quality level and translate it into a reasonable transmission bit rate so that the deployments can be scalable and reliable. Immersive and haptic systems can help the user feel more responsive regarding the control flow involved in the remote operation, but will also require of being enhanced by AI architectures to be fast and efficient.

### 5.2. Data Integrity and Security of Control Protocol Key Performance Indicators

It is crucial to highlight the vital role of digital signatures in ensuring the authenticity and integrity of data stored in modern systems. In the context of vehicles, where various parameters such as location, speed, and environmental conditions are monitored, the risk of tampering with these records poses significant challenges. By employing digital signatures, each vehicle can generate a unique identifier for its data prior to storage, enabling the prompt detection of unauthorized modifications. This approach enhances security and reduces potential safety risks associated with falsified data. Decentralized and immutable blockchain technologies offer promising solutions to these challenges, though they must address issues of scalability and high computational costs. A prototype integrating a blockchain system with replicated nodes into a real teleoperated control system, deployed across three European projects, has demonstrated the feasibility of further advanced developments.

### 5.3. Teleoperated Driving Under 5G-Advanced Systems

L4S (Low-Latency, Low-Loss, Scalable throughput) technologies in 5G-Advanced networks introduce sophisticated mechanisms for application-level bandwidth control, enabling dynamic and intelligent resource allocation. A practical application of this innovation is the adaptive adjustment of video bit rate for cameras deployed in TOD setups. By leveraging ECN counters, edge applications can monitor and manage traffic across different 5G network slices, ensuring efficient utilization of bandwidth while preventing packet loss. This approach promotes equitable bandwidth distribution among User Equipment (UE) operating within the same slice, fostering a more democratic and balanced use of network resources. Furthermore, the ability to dynamically adjust video bit rate based on real-time traffic conditions enhances the reliability and scalability of TOD systems, which are highly sensitive to latency and network stability. An experimental deployment has successfully demonstrated the feasibility of this method, showcasing its potential to optimize network performance while maintaining the stringent requirements of latency-critical applications. As 5G-advanced networks continue to evolve, L4S technologies are poised to play a pivotal role in supporting innovative use cases that demand both high efficiency and robust traffic control.

### 5.4. Contributions and Future Directions

Beyond empirical measurements and deployment descriptions, this paper makes three concrete contributions. First, we present a unified end-to-end XR teleoperation architecture that combines video encoding strategies, immersive rendering, and network function design to meet TOD latency and reliability constraints. Second, we provide systematic QoS/QoE experiments across three real-world 5G testbeds, quantifying trade-offs between bit rate, latency, and task-level driving performance and deriving practical operating envelopes. Third, we propose and evaluate deployment strategies—including planar rendering, adaptive encoding, and prioritized network slicing—that jointly optimize operator perception, bandwidth usage, and safety. These contributions aim to bridge component-level research into deployable TOD systems.

As technology continues to advance, the need for robust mechanisms to ensure data integrity and sophisticated communication protocols is becoming increasingly evident. The integration of AI, blockchain, and 6G technologies represents only the beginning of a vast array of potential applications. Future research should prioritize the development of more advanced algorithms for real-time anomaly detection, improving the energy efficiency of interconnected systems, and exploring innovative methods to enhance user privacy while maintaining system security.

The transformative impact of cutting-edge technologies necessitates addressing challenges such as data integrity, secure communication, and autonomous control. These efforts will pave the way for a future where TOD deployments play an increasingly central role in shaping everyday life. The advancements discussed here highlight the importance of interdisciplinary collaboration and establish foundational pillars for future research.

Hybrid autonomous and supervised remote driving offers a promising pathway for the seamless deployment of autonomous driving systems. This innovative approach combines the strengths of autonomous systems with remote control capabilities, aiming to enhance safety, efficiency, and adaptability across diverse driving scenarios. By merging AI-driven decision-making with real-time human oversight, hybrid systems provide a dynamic solution that balances autonomy with human intervention when necessary.

The future of hybrid autonomous driving depends on continuous research and development in key areas:

1. AI Enhancements: Advancing machine learning algorithms to enable more nuanced decision-making, thereby reducing reliance on human intervention.
2. Human–Machine Interfaces: Creating intuitive interfaces that facilitate effective interaction between operators and AI systems during remote control.
3. Regulatory Frameworks: Establishing comprehensive regulations to support the deployment of hybrid autonomous vehicles while addressing ethical and legal considerations.

In conclusion, hybrid autonomous driving represents a significant leap forward in automotive technology, offering a balanced approach that integrates advanced AI with reliable remote control systems. This innovation enhances safety, adaptability, and efficiency across various applications. As research continues to progress, overcoming current challenges will enable broader adoption and further advancements in mobility solutions [47].

## Figures and Tables

**Table 1:** EMLID REACH M2 positioning accuracies.

Accuracy Description	Value	Error
Static Horizontal	4 mm	0.5 ppm
Static vertical	8 mm	1 ppm
Kinematic horizontal	7 mm	1 ppm
Kinematic vertical	14 mm	1 ppm

**Table 2:** Basic and extended statistics for control latency in digital twin scenarios.

Metric	Basic Stats								Extended Stats	
	Count	Mean	Std	Min	25%	50%	75%	Max	MAD	Skewness
Latency (ns)	1624	9730.89	4888.61	6294	7247.75	8257	9924.5	42,520	1160	3.49244

**Table 3:** Description of latency-related variables.

Variable Name	Description	Units
$T_{c}$	Time to capture one video frame	ms
$T_{e}$	Time to encode one video frame	ms
$B_{t}$	Transmission bit rate	Mbps
$B_{e}$	Encoded video bit rate	Mbps
FPS	Encoded video frames per second	1/s
$T_{d}$	Time to decode one video frame	ms
$T_{r}$	Time to render one video frame	ms
$T_{\mathrm{RTT}}$	Round-trip time (RTT)	ms
$T_{h}$	Human reaction time	ms
$T_{x}$	Car command execution delays	ms

**Table 4:** Basic and extended statistics for selected latency and bandwidth measurements.

Metric	Basic Stats				Extended Stats		
	Count	Mean	Std	Min	MAD	Skewness	*p*-Value
MJPEG_H.264 (ms)	119	207.311	25.947	105.000	13.000	0.943	5.722 × 10^−109^
MJPEG_H.264 (Mbps)	119	3.935	0.093	3.812	0.004	−0.539	3.216 × 10^−194^
MJPEG (ms)	119	126.025	16.875	95.000	10.000	2.459	1.453 × 10^−105^
MJPEG (Mbps)	119	57.674	0.296	57.072	0.195	0.443	2.666 × 10^−272^

**Table 5:** Pearson correlation coefficients for selected metrics.

	VMAF	PSNR	P1204_3_MOS	COVER_TSC	COVER_ASC	COVER_FSC	SI_PCT	VCA_E_PCT	VCA_H_PCT
LD_PCT	0.7966	0.7212	0.7584	0.5157	0.6144	0.5652	0.7092	0.6305	0.5650
CARS_PCT	0.6305	0.5296	0.6065	0.5484	0.6380	0.5989	0.5595	0.5028	0.5585

**Table 6:** *p*-value coefficients for selected metrics.

	VMAF	PSNR	P1204_3_MOS	COVER_TSC	COVER_ASC	COVER_FSC	SI_PCT	VCA_E_PCT	VCA_H_PCT
LD_PCT	0	1.80 × 10^−301^	0	3.58 × 10^−128^	1.94 × 10^−195^	4.29 × 10^−159^	3.24 × 10^−287^	9.08 × 10^−209^	7.69 × 10^−159^
CARS_PCT	1.77 × 10^−205^	4.11 × 10^−134^	3.81 × 10^−186^	1.07 × 10^−145^	7.26 × 10^−212^	2.13 × 10^−180^	7.93 × 10^−153^	4.64 × 10^−119^	4.64 × 10^−152^

**Table 7:** Confidence intervals for selected metrics.

	VMAF	PSNR	P1204_3_MOS	COVER_TSC	COVER_ASC	COVER_FSC	SI_PCT	VCA_E_PCT	VCA_H_PCT
LD_PCT (upper)	0.805	0.732	0.768	0.532	0.629	0.581	0.720	0.644	0.580
LD_PCT (lower)	0.788	0.710	0.748	0.499	0.600	0.549	0.698	0.616	0.549
CARS_PCT (upper)	0.644	0.546	0.621	0.565	0.652	0.614	0.575	0.520	0.574
CARS_PCT (lower)	0.616	0.513	0.592	0.532	0.624	0.584	0.543	0.485	0.542

## Data Availability

Data related to video quality metrics and object detection measurements is publicly available at https://github.com/nachobfrupm/TOD_QualityVideoEncoding (accessed on 21 December 2025).

## Abbreviations

    The following abbreviations are used in this manuscript:

AI	Artificial Intelligence
AGV	Automated Guided Vehicle
AQM	Active Queue Management
AR	Augmented Reality
CCAM	Cooperated, Connected, and Automated Mobility
CDF	Cumulative distribution function
COVER	Comprehensive Video Quality Evaluator
E2E	End-To-End
FPS	Frames Per Second
ECN	Explicit Congestion Notification
EVCA	Enhanced Video Complexity Analyser
FEC	Forward Error Correction
GPS	Global Positioning System
GPT	Generative Pre-trained Transformer
GPU	Graphics Processing Unit
KPI	Key Performance Indicator
L4S	Low Latency, Low Loss, Scalable Throughput
LLM	Large Language Model
LTE	Long-Term Evolution
MJPEG	Motion JPEG
MPC	Multi-Party Computation
MEC	Multi Edge Computing
mMTC	massive Machine-Type Communication
PSNR	Peak Signal-to-Noise Ratio
PRB	Physical Resource Block
RAN	Radio Access Network
RTK	Real-time kinematic positioning
RTP	Real-time Transport Protocol
RTT	Round-Trip Time
SINR	Signal-to-Interference-plus-Noise Ratio
SLA	Service Level Agreement
TCP	Transmission Control Protocol
TOD	teleoperated Driving
QoE	Quality of Experience
QoS	Quality of Service
ROS	Robot Operating System
UDP	User Datagram Protocol
URLLC	Ultra-Reliable Low Latency
VCA	Video Content Analysis
VMAF	Video Multimethod Assessment Fusion
VQA	Video Quality Assessment
VR	Virtual Reality
XR	eXtended Reality
YOLO	You Only Look Once
